# A benchmarking study of individual somatic variant callers and voting-based ensembles for whole-exome sequencing

**DOI:** 10.1093/bib/bbae697

**Published:** 2025-01-18

**Authors:** Arnaud Guille, José Adélaïde, Pascal Finetti, Fabrice Andre, Daniel Birnbaum, Emilie Mamessier, François Bertucci, Max Chaffanet

**Affiliations:** Predictive Oncology Laboratory, Marseille Research Cancer Center, INSERM U1068, CNRS U7258, Institut Paoli-Calmettes, Aix-Marseille University, Equipe labellisée « Ligue Nationale Contre le Cancer », 13009 Marseille, France; Predictive Oncology Laboratory, Marseille Research Cancer Center, INSERM U1068, CNRS U7258, Institut Paoli-Calmettes, Aix-Marseille University, Equipe labellisée « Ligue Nationale Contre le Cancer », 13009 Marseille, France; Predictive Oncology Laboratory, Marseille Research Cancer Center, INSERM U1068, CNRS U7258, Institut Paoli-Calmettes, Aix-Marseille University, Equipe labellisée « Ligue Nationale Contre le Cancer », 13009 Marseille, France; Department of Medical Oncology, Gustave Roussy, University Paris-Saclay, 94805 Villejuif, France; Predictive Oncology Laboratory, Marseille Research Cancer Center, INSERM U1068, CNRS U7258, Institut Paoli-Calmettes, Aix-Marseille University, Equipe labellisée « Ligue Nationale Contre le Cancer », 13009 Marseille, France; Predictive Oncology Laboratory, Marseille Research Cancer Center, INSERM U1068, CNRS U7258, Institut Paoli-Calmettes, Aix-Marseille University, Equipe labellisée « Ligue Nationale Contre le Cancer », 13009 Marseille, France; Predictive Oncology Laboratory, Marseille Research Cancer Center, INSERM U1068, CNRS U7258, Institut Paoli-Calmettes, Aix-Marseille University, Equipe labellisée « Ligue Nationale Contre le Cancer », 13009 Marseille, France; Medical Oncology, Institut Paoli-Calmettes, 13009, Marseille, France; Predictive Oncology Laboratory, Marseille Research Cancer Center, INSERM U1068, CNRS U7258, Institut Paoli-Calmettes, Aix-Marseille University, Equipe labellisée « Ligue Nationale Contre le Cancer », 13009 Marseille, France

**Keywords:** NGS, somatic, variant caller, benchmark, ensemble, combination, voting

## Abstract

By identifying somatic mutations, whole-exome sequencing (WES) has become a technology of choice for the diagnosis and guiding treatment decisions in many cancers. Despite advances in the field of somatic variant detection and the emergence of sophisticated tools incorporating machine learning, accurately identifying somatic variants remains challenging.

Each new somatic variant caller is often accompanied by claims of superior performance compared to predecessors. Furthermore, most comparative studies focus on a limited set of tools and reference datasets, leading to inconsistent results and making it difficult for laboratories to select the optimal solution. Our study comprehensively evaluated 20 somatic variant callers across four reference WES datasets. We subsequently assessed the performance of ensemble approaches by exploring all possible combinations of these callers, generating 8178 and 1013 combinations for single-nucleotide variants (SNVs) and indels, respectively, with varying voting thresholds. Our analysis identified five high-performing individual somatic variant callers: Muse, Mutect2, Dragen, TNScope, and NeuSomatic. For somatic SNVs, an ensemble combining LoFreq, Muse, Mutect2, SomaticSniper, Strelka, and Lancet outperformed the top-performing caller (Dragen) by >3.6% (mean F1 score = 0.927). Similarly, for somatic indels, an ensemble of Mutect2, Strelka, Varscan2, and Pindel outperformed the best individual caller (Neusomatic) by >3.5% (mean F1 score = 0.867). By considering the computational costs of each combination, we were able to identify an optimal solution involving four somatic variant callers, Muse, Mutect2, and Strelka for the SNVs and Mutect2, Strelka, and Varscan2 for the indels, enabling accurate and cost-effective somatic variant detection in whole exome.

## Introduction

Somatic mutations play a critical role in cancer development and progression [1]. Whole-exome sequencing (WES) has become a powerful and affordable tool for both cancer diagnosis and therapeutic strategy definition [2]. However, our ability to distinguish the somatic mutations from sequencing artifacts and polymorphisms remains limited because of several pitfalls, and this has clinical consequences.

These pitfalls are due to tumor intrinsic characteristics, sample/libraries preparation and sequencing, and selection of inappropriate software, which can generate interpretation mistakes. Indeed, cellular and molecular heterogeneity (subclonal architecture) within a tumor contaminated by normal tissue (stroma) hinders the detection of somatic variants with low allelic frequencies (VAF) [3, 4]. Sample handling and library preparation and sequencing [5, 6] may generate false-positive variants at low VAF that can be mistaken for true somatic mutations. Another critical step in the process of the somatic mutations detection is the selection of appropriate variant callers software.

The advent of next-generation sequencing has led to a vast array of software tools (https://usegalaxy.org/). Choosing the most appropriate one requires bioinformatic expertise, especially since these tools have variable performances [7], which can significantly impact downstream analyses and influence clinical decision. The emergence of machine learning and deep learning in the area of variant detection showed promises for improved accuracy [8, 9], but the results have not yet met the expectations and their implementation in production settings faces hurdles. Such models suffer from data dependency with overfitting [10]. Training them requires to gather massive high-quality datasets [11], which can be expensive and time consuming. Finally, these models act like “black boxes” making them difficult to interpret and fine-tune for non-experts [12].

Previous studies have already reported benchmarks of various widely used somatic variant callers in several reference datasets. The International Cancer Genome Consortium (ICGC) and The Cancer Genome Atlas (TCGA) DREAM somatic mutation calling challenge proposed to identify the most accurate pipeline to detect somatic variants in synthetic tumors computationally created from real cancer samples [13]. The SEQC2 consortium sequenced a well-known tumor cell line and its normal counterpart under different conditions and with orthogonal technologies (Illumina, PacBio, AmpliSeq, Ion Torrent) [14]. This provided the community with a comprehensive set of reference variants, which were then used to benchmark several analytic tools [15]. Other studies used samples from the platinum genomes, which consist in a reference dataset with variants validated by inheritance over three generations to create virtual tumor–normal paired samples with different levels of contamination and sequencing depth [16, 17]. This allowed the study of the impact of purity and depth on somatic variant detection accuracy.

Despite numerous studies [11, 13, 17–22], limitations remain due to the narrow scope of previous benchmarks. Indeed, the majority of them focused on a limited set of tools in a specific dataset. However, the tool performance can vary significantly across reference datasets, highlighting the need for evaluations across multiple datasets. Currently, no single study offers a comprehensive benchmark for an exhaustive list of somatic variant callers across diverse datasets. Combining multiple somatic variant callers with ensemble approach and decision-voting algorithms has shown promise [10, 20–22], but uncertainties remain regarding the optimal set and number of tools to include.

Our study aimed at comprehensively compare the performances of 20 somatic variant callers across several datasets. The comparison encompassed different algorithm types (classic and deep learning) and license models (non-commercial and commercial). Four WES reference datasets with intrinsic features were used for evaluation. The performances of individual tools were compared to an ensemble-based approach. Furthermore, post-alignment procedures, computational time, and memory usage were also assessed to identify a set of tools and strategies that could be readily applied in a laboratory setting.

## Methods

### Presentation of datasets

#### ICGC-TCGA DREAM challenge data

Stage 3 dataset (NGV3) is a synthetic paired tumor–normal dataset derived from the HCC1143 cell line [13]. Briefly, the HCC1143 cell line was sequenced at 80x (WGS), then the bam file was split into two subsets to simulate the tumor and its normal counterpart. The tumor sample with multiple subclones was obtained by computationally adding mutations at different frequencies (50%, 33%, and 20%) in one subset. Exome data for this dataset were retrieved from https://github.com/bcbio/bcbio-nextgen. This dataset included 474 and 464 true-positive somatic single-nucleotide variants (SNVs) and insertions/deletions (indels), respectively. For this dataset, we restricted the analysis to the exome regions available at https://github.com/AstraZeneca-NGS/reference_data/blob/master/hg19/bed/Exome-NGv3.bed.

#### SEQC2 dataset

The SEQC2 consortium generated a comprehensive resource of paired tumor/normal reference samples [14]. This resource was created by sequencing the HCC1395 triple-negative breast cancer (TNBC) cell line [23], and the cognate HCC1395BL B lymphocyte–derived normal cell line using various sequencing technologies and platforms across multiple centers. Then, several bioinformatics pipelines were employed to establish a high-confidence reference call set of true somatic variants. For our benchmarks, we used WES datasets SRR7890883 (253x) and SRR7890874 (300x), sequenced with Illumina HiSeq 4000. This dataset included 1160 and 50 true-positive somatic SNVs and indels, respectively. For validation, we used an additional sample (SRR7890879) with a sequencing depth of 76x, obtained from a different platform (Fudan University). This sample provided an independent dataset to assess the reproducibility of our findings. For this dataset, we restricted the analysis to the high-confidence regions available at ftp-trace.ncbi.nlm.nih.gov/ReferenceSamples/seqc/Somatic_Mutation_WG/release/latest/.

#### PERMED-01 dataset

From 120 clinical breast cancer samples with whole-exome and targeted sequencing (WES and t-NGS) applied in our PERMED-01 study [24, 25], 36 were selected based on their mean sequencing depth >150x. These samples had a mean sequencing depth of 206x. A t-NGS approach using three different panels covering 395, 494, and 560 genes was used to create the ground truth set of somatic mutations.

To reduce the computational time and obtain a dataset with a sufficient number of somatic mutations for benchmarking, WES data from these 36 tumor samples and their matched normal controls were computationally merged with PICARD tools.

To preserve the original allele frequencies of the somatic mutations in the merged reads obtained from all tumor samples, a two-step downsampling procedure was applied as follows:

(i) Outside the regions containing somatic mutations, both tumor and normal samples were downsampled to 1/36 of their original depth.
(ii) Within the regions containing somatic mutations, reads were specifically extracted from the corresponding tumor/normal paired samples where the somatic mutation occurred.

This dataset included 175 and 38 true-positive somatic SNVs and indels, respectively.

To prevent multiallelic site at true somatic points, when several samples had the same somatic mutations, the sample with the highest VAF was kept.

For this dataset, we restricted the analysis to the regions common between the different panels.

#### HCC1143 dataset

Whole-exome profiles of the HCC1143 TNBC cell line [23] and the cognate HCC1143BL B lymphocyte–derived normal cell line were retrieved from the Sequence Read Archive (SRA) (https://www.ncbi.nlm.nih.gov/sra). Samples SRR6438473 and SRR6438475 were used for our benchmarks as two whole exomes sequenced at respectively 245x and 317x with Illumina HiSeq 4000. The truth set of somatic mutations were downloaded from https://docs.icgc-argo.org/. This dataset included 257 true-positive somatic SNVs and no indel. For this dataset, we restricted the analysis to the regions available at https://support.illumina.com/downloads/nextera_rapid_capture_exome_unique_intervals_file.html.

### Alignment and variant calling

The raw reads were aligned using bwa mem to the human genome reference (hg19 or hg38) corresponding to the reference used for the true set of somatic variants for each dataset [26]. Duplicated reads were marked with sambamba, and base quality score recalibration (BQSR) was done with GATK4 [27, 28]. Influence of post-alignment procedures on variant calling was evaluated using four different strategies (bwa; bwa + deduplication; bwa + BQSR; bwa + deduplication + BQSR).

A total of 20 variants callers (18 for SNVs; 15 for indels) were chosen for the evaluation based on a comprehensive literature review. We prioritized widely used tools such as Lancet [29], LoFreq [30], Muse [31], Mutect [32], Mutect2 [33], Scalpel [34], Seurat [35], SomaticSniper [36], Strelka [37], Vardict [38], Varscan2 [39], Shimmer [40], and Virmid [41] encompassing various algorithmic approaches including haplotype, heuristic threshold, and joint genotype analyses. While FreeBayes and Pindel were not initially designed for somatic analysis [42, 43], they were also used with filtering strategies between tumor and normal samples for identifying somatic variants. Additionally, we considered recent deep learning–based tools such as DeepSomatic [44], NeuSomatic, and VarNet [8, 45]. Finally, to assess the performance of commercially available options, we included Dragen (Illumina) and TNScope (Sentieon) based on their evaluations in published benchmarks [46–48]. The full list of individual somatic variant callers is summarized in Table 1.

**Table 1:** Variant callers used in the study

**Type of algorithm**	**Variant caller**	**Version**	**Type of variant**	**Keep for ensemble**	**Link**	**Ref**
Allele frequency analysis	Lofreq	2.1.5	SNV, INDEL	Yes	https://github.com/CSB5/lofreq/raw/master/dist/lofreq_star-2.1.5.tar.gz	[30]
Allele frequency analysis	Strelka	2.9.2	SNV, INDEL	Yes	https://github.com/Illumina/strelka/releases/download/v2.9.2/strelka-2.9.2.centos6_x86_64.tar.bz2	[37]
Haplotype analysis	FreeBayes	1.3.4	SNV, INDEL	Yes	https://github.com/freebayes/freebayes/releases/download/v1.3.4/freebayes-1.3.4-linux-static-AMD64.gz	[42]
Haplotype analysis	Mutect	1.1.7	SNV	Yes	gs://gatk-software/package-archive/mutect/mutect-1.1.7.jar.zip	[32]
Haplotype analysis	Mutect2	4.2.2.0	SNV, INDEL	Yes	https://github.com/broadinstitute/gatk/releases/download/4.2.2.0/gatk-4.2.2.0.zip	[33]
Heuristic threshold	Pindel	1.1	INDEL	Yes	https://github.com/genome/pindel/archive/70c1bb4a75503da39e206e02178fe3d8a0afdf81.tar.gz	[43]
Heuristic threshold	Shimmer	0.2	SNV	Yes	https://github.com/nhansen/Shimmer	[40]
Heuristic threshold	Vardict	1.8.3	SNV, INDEL	Yes	https://github.com/AstraZeneca-NGS/VarDictJava.git	[38]
Heuristic threshold	Varscan2	2.3.9	SNV, INDEL	Yes	https://sourceforge.net/projects/varscan/files/VarScan.v2.3.9.jar	[39]
Joint genotype analysis	Lancet	1.1.0	SNV, INDEL	Yes	https://github.com/nygenome/lancet	[29]
Joint genotype analysis	Seurat	2.5	SNV, INDEL	Yes	https://github.com/tgen/seurat	[35]
Joint genotype analysis	SomaticSniper	1.0.5.0	SNV	Yes	https://github.com/genome/somatic-sniper	[36]
Joint genotype analysis	Virmid	1.1.0	SNV	Yes	https://sourceforge.net/projects/virmid/files/virmid-1.1.0.tar.gz	[41]
Markov chain model	Muse	2.0	SNV	Yes	https://github.com/wwylab/MuSE	[31]
Microassembly	Scalpel	0.5.4	INDEL	Yes	https://sourceforge.net/projects/scalpel/files/scalpel-0.5.4.tar.gz	[34]
Neural network	DeepSomatic	1.7.0	SNV, INDEL	No	https://github.com/google/deepsomatic	[44]
Neural network	NeuSomatic	0.2.1	SNV, INDEL	No	https://github.com/bioinform/neusomatic	[8]
Neural network	VarNet	1.1.0	SNV, INDEL	No	https://github.com/skandlab/VarNet	[45]
Haplotype analysis	Dragen^a^	4.2.7	SNV, INDEL	No	https://basespace.illumina.com	[46]
Haplotype analysis	TNScope^a^	202308.02	SNV, INDEL	No	https://www.sentieon.com/	[47]

^a^Commercial license (trial version)

Detailed command lines and parameters used for each caller are provided in the Supplementary method file.

Base space (Illumina) was used to run Dragen-somatic software with default parameters without alignment option.

### Ensemble approach

We initially conducted somatic variant calling independently for each of the 20 callers. The resulting VCF files were then merged into a single VCF file. To identify the most effective ensemble approach, we evaluated all possible combinations of variant callers, ranging from 2 to 13 tools for SNVs and 2 to 11 tools for indels. For this analysis, we did not keep the two commercial tools (Dragen and TNScope) and the three deep-learning tools (DeepSomatic, NeuSomatic, and VarNet).

The total number of combinations was computed using the following formula:


$$ C{\displaystyle \begin{array}{c}p\\{}n\end{array}}=\frac{n!}{p!\left(n-p!\right)} $$

where *n* is the total number of tools and *p* is the combination size.

For each combination size (*p* tools), we further evaluated the impact of the minimum voting threshold. This threshold determines the minimum number of callers that must agree on a variant for it to be considered a positive call. We tested thresholds ranging from 1 (any tool) to *p* (all participating tools agree).

### Evaluation and metrics

For the evaluation of performances, the somatic variants reported in the truth set were defined as the positive set (P).

Several metrics were used to assess the performance of the variant callers. The different levels of a caller’s accuracy are as follows:

True positive (TP) = number of correctly detected true variants from the truth set;

False positive (FP) = number of variants identified by a caller that are not present in the truth set;

False negative (FN) = number of true variants from the truth set that are missed by a caller.

Based on these definitions, we calculated the following metrics:

Recall, sensitivity, or true-positive rate (TPR) = TP / (TP + FN). This metric indicates the proportion of true variants that are correctly called by the tool;

Precision or positive predictive value (PPV) = TP / (TP + FP). This metric reflects the proportion of variants called by the tool that are truly present in the sample;

F1 score = (2*TP) / (2*TP + FP + FN). The F1 score balances precision and recall, providing a more comprehensive assessment of a caller’s performance.

Due to the inherent class imbalance between positive (true variants) and negative (non-variant sites) classes, accuracy was not used as a performance metric.

Variance was computed according to the following formula: Var(X) = E[(X − E[X])^2^].

CPU time (real time) and memory usage (resident set size) for both individual somatic variant callers and ensemble approaches were collected with Snakemake benchmark option [49]. For the ensemble approach, the CPU times for the set of tools included in a combination were summed.

## Results

### Description of the four reference datasets used for evaluations

Four datasets were used for the evaluation of individual variant callers and ensemble approaches (Table 2). First, NGV3 dataset was computationally derived from the WGS dataset of a cell line (HCC1143). This dataset mimics the clonal architecture of real tumors by including true-positive somatic SNVs (*n* = 474) and indels (*n* = 464) with varying VAFs (50%, 33%, and 20%). Second, SEQC2 WES dataset (HCC1395) included 1160 and 50 true-positive somatic SNVs and indels with a median VAF at 10% (min = 0%, max = 100%), respectively. Third, the WES dataset of the HCC1143 TNBC breast cell line included 257 true-positive somatic SNVs and no indel with a median VAF at 10% (min = 0%, max = 100%). Fourth, the PERMED-01 dataset was the only one derived from breast cancer patients. It included 175 and 38 true-positive somatic SNVs and indels with a median VAF at 20% (min = 2%; max = 60%), respectively (Fig. S1).

**Table 2:** Reference datasets used in the study

**Name**	**Platform**	**Sample**	**Link**	**Number of SNVs**	**Number of indels**	**Coverage**	**Duplication rate (%)**	**Contamination**	**Error rate**
NGV3	*In silico*	HCC1143	https://github.com/bcbio/bcbio-nextgen	474	464	40x	4.85	1.08E−03	2.17E−04
SEQC2	Illumina HiSeq 4000	HCC1395 (WES_IL_T_1)	https://www.ncbi.nlm.nih.gov/sra/?term=SRR7890883	1160	50	253x	45.03	9.13E−02	2.80E−03
PERMED-01	Illumina	Metastatic breast cancer samples	https://ega-archive.org/studies/EGAS00001003290	175	38	206x	0.87	6.20E−01	2.20E−02
HCC1143	Illumina HiSeq 2500	HCC1143	https://www.ncbi.nlm.nih.gov/sra/?term=SRR6438473	257	0	245x	30.66	1.94E−03	4.01E−04
SEQC2-FD	Illumina HiSeq 4000	HCC1395 (WES_FD_T_1)	https://www.ncbi.nlm.nih.gov/sra/SRX4728489	1160	50	76x	23.49	4.73E−03	6.67E−04

### Comparison of performances of somatic variant calling softwares

To evaluate the performance of widely used somatic variants callers, the F1 scores obtained across four different reference datasets were compared.

**Figure 1:**
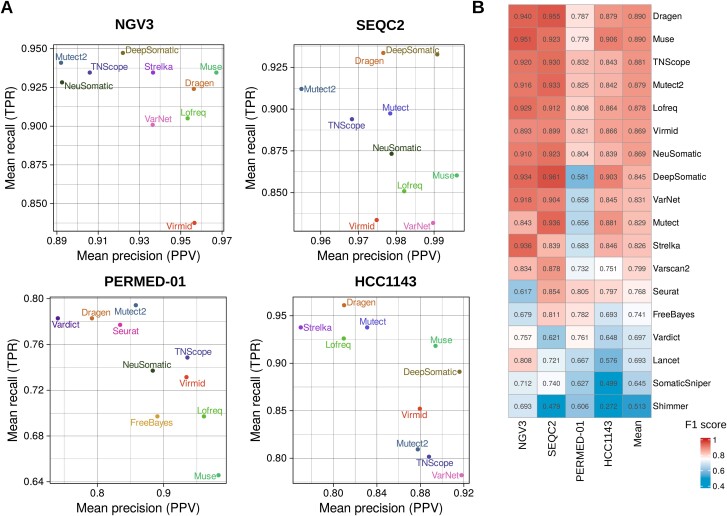
Performance evaluation for 18 individual somatic variant callers in the four datasets for the SNVs. (A) For each dataset, recall (TPR) and precision (PPV) were calculated for the top 10 somatic variant callers. (B) Heatmap showing F1 scores of the somatic variant callers in the four datasets. Tools were ranked based on their mean F1 scores.

Dragen achieved the highest mean F1 score of 0.890 (min = 0.787, max = 0.955) for somatic SNVs detection across the four datasets. Muse and TNScope secured the second and third positions, with mean F1 scores of 0.890 and 0.881, respectively. Mutect2 showed solid performances closed to the top three with a mean F1 score of 0.879 (min = 0.825, max = 0.933). Among the deep-learning models, NeuSomatic achieved the best result for the somatic SNVs, with a mean F1 score of 0.869 (min = 0.804, max = 0.923) (Fig. 1**,**  Table S1).

**Figure 2:**
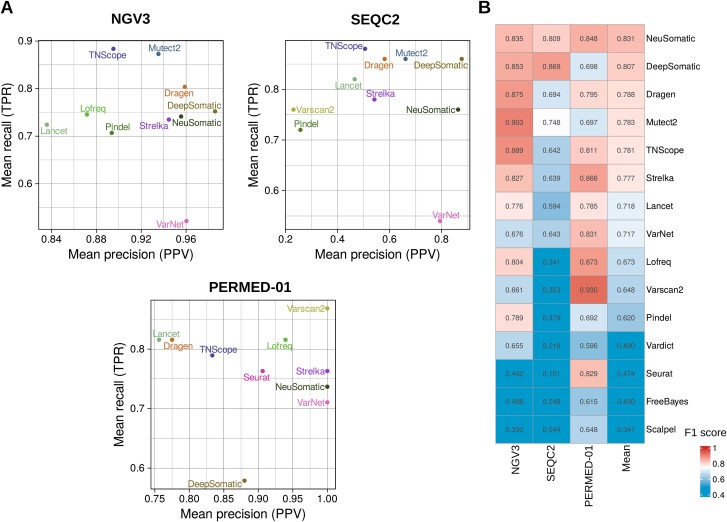
Performance evaluation for 15 individual somatic variant callers in the three datasets for the indels. (A) For each dataset, recall (TPR) and precision (PPV) were calculated for the top 10 somatic variant callers. (B) Heatmap showing F1 scores of the somatic variant callers in the four datasets. Tools were ranked based on their mean F1 scores.

For somatic indels detection, NeuSomatic achieved the best results, with a mean F1 score of 0.831 (min = 0.809; max = 0.849), followed by DeepSomatic and Dragen achieving mean F1 scores of 0.807 and 0.788, respectively (Fig. 2, Table S2).

When considering both somatic SNVs and indels (*n* = 13 variants callers), NeuSomatic, Dragen, and TNScope emerged as the top performers, achieving mean global F1 scores of 0.849, 0.839, and 0.831, respectively. Notably, these three softwares demonstrated strong performances in detecting both somatic SNVs and indels. Mutect2 followed closely behind with a mean global F1 score of 0.831.

### Variation of performance across the reference datasets

**Figure 3:**
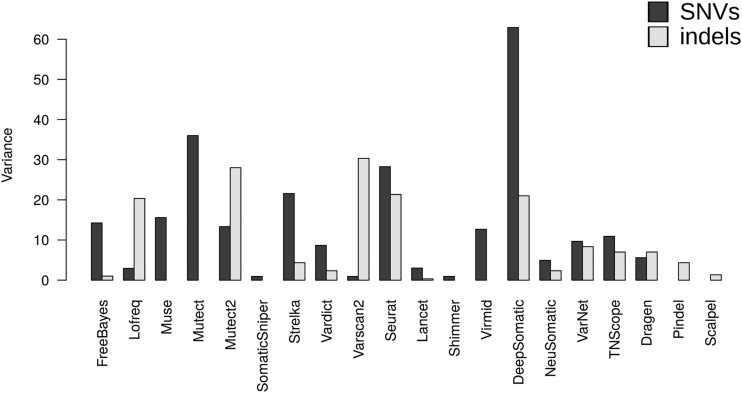
Variation of performance for 20 individual somatic variant callers for the SNVs and indels. Variant callers were ranked according to the mean F1 scores. Then, variance of the ranks was calculated for the SNVs and indels separately.

While some individual variant callers exhibited relative stability across the datasets, some callers displayed significant variations in rank depending on the dataset (Fig. 3). For the SNVs, Mutect, Strelka, and Seurat displayed a high variance in their ranking. For example, Mutect achieved top-tier performance (F1 score ≥ 0.94) in the SEQC2 and HCC1143 datasets, but its performance dropped in NGV3 and PERMED-01 datasets. For the indel detection, a high variance in the ranking was observed for Mutect2, Varscan2, and Seurat. For example, Mutect2 achieved the best ranking in the NGV3 dataset and the second place in the SEQC2 data, but failed in the PERMED-01 dataset (rank = 11), principally due to a high number of false negatives with the “clustered event” filter, which reject some true variants. Interestingly, NeuSomatic, a deep learning–based caller, demonstrated better and consistent performance across all four datasets. However, DeepSomatic and VarNet, two other deep-learning methods, showed poor performance within the PERMED-01 dataset in the detection of somatic SNVs. It was particularly pronounced for DeepSomatic, which demonstrated excellent results in the other three datasets. After investigation, we noticed that this was due to a high number of false-positive somatic variants, which were actually germline variants. These observations highlight the importance of validating variant caller performance across diverse datasets to ensure generalization.

### Evaluation of the ensemble approach

To compare the ensemble approach against commercially available softwares and tools that use more complex methods like deep learning, we excluded the two commercial somatic variant callers and the three deep learning–based callers, leaving 15 callers for the ensemble approach evaluation.

To identify the most effective ensemble approach, we evaluated all possible combinations of the 15 pre-selected variant callers (13 for the SNVs, 10 for the indels). This resulted in a comprehensive analysis of 8178 and 1013 different combinations for SNVs and indels, respectively. For each combination, we tested a range of thresholds (1 to *n* callers) to determine the minimum number of callers required to agree on a variant call for it to be considered a positive finding. This resulted in the evaluation of 53 235 and 5110 combinations with varying voting thresholds for SNVs and indels, respectively.

Our comprehensive evaluation revealed some general trends for the ensemble approach. For both SNVs and indels, performance peaked at an optimal number of participating tools, typically between four and six across most datasets (Figs. S2–S5). This trend was also observed for the voting threshold. However, the specific combination of tools yielding the best performance varied across datasets. For instance, in the NGV3 dataset, the combination of Lofreq, Muse, Mutect2, and Virmid with a minimum of two agreeing votes formed the top-performing ensemble for SNVs, while the combination of Mutect2, Strelka, Vardict, Pindel, Varscan2, and Lancet with a minimum of two agreeing votes achieved the best results for indels. In contrast, the SEQC2 dataset showed optimal performance with ensembles containing Muse, Mutect2, Vardict, and Varscan2 with a minimum of two agreeing votes for SNVs, while LoFreq, Mutect2, Strelka, Vardict, and Seurat with a minimum of four agreeing votes achieved the best results for indels (Tables S3–S4).

To identify a consistently high-performing ensemble across diverse datasets, we calculated the average F1 score for each combination of tools. Notably, results showed that for somatic SNVs, an ensemble combining Lofreq, Muse, Mutect2, SomaticSniper, Strelka, and Lancet with a minimum of three agreeing votes achieved the best average F1 score equal to 0.927 (Table S3). This represents a significant 3.7% improvement compared to the best result obtained by Dragen (mean F1 score = 0.890) as single caller. Similarly, for somatic indel detection, the ensemble containing Mutect2, Strelka, Pindel, and Varscan2 with a two-vote threshold achieved the best average performance (F1 score = 0.867), surpassing the best deep-learning model single-caller NeuSomatic (mean F1 score = 0.831) by 3.6% (Table S4).

When considering both somatic SNVs and indels, the best ensemble performance was achieved with a combination of Mutect2, Strelka, and Varscan2, requiring a minimum of two agreeing votes (Table S5). This ensemble yielded a mean global F1 score of 0.885. Notably, this represents a 4% improvement in performance compared to the best result obtained by any single variant caller (mean F1 score = 0.849).

### Evaluation of performance with different post-alignment processing

To evaluate the influence of different post-alignment procedures, we evaluated four different approached (bwa only; bwa + deduplication; bwa + BQSR; bwa + deduplication + BQSR) across the four reference datasets.

Our analysis revealed dataset-specific trends. Datasets with lower duplication rates, such as NGV3 and PERMED-01, were less affected by deduplication and BQSR. Conversely, in the SEQC2 and HCC1143 datasets, deduplication significantly increased the F1 scores for most somatic variant callers, particularly for SNVs. The impact of BQSR was more limited, with a noticeable benefit for Strelka but less pronounced effects on other tools like Muse, Mutect2, and DeepSomatic. (Tables S1–S2**,**  Figs. S6–S7).

### Comparison of processing time and memory consumption

To compare the computational cost for both individual somatic variant callers and ensemble approaches, CPU times and memory usage reported by Snakemake were collected.

**Figure 4:**
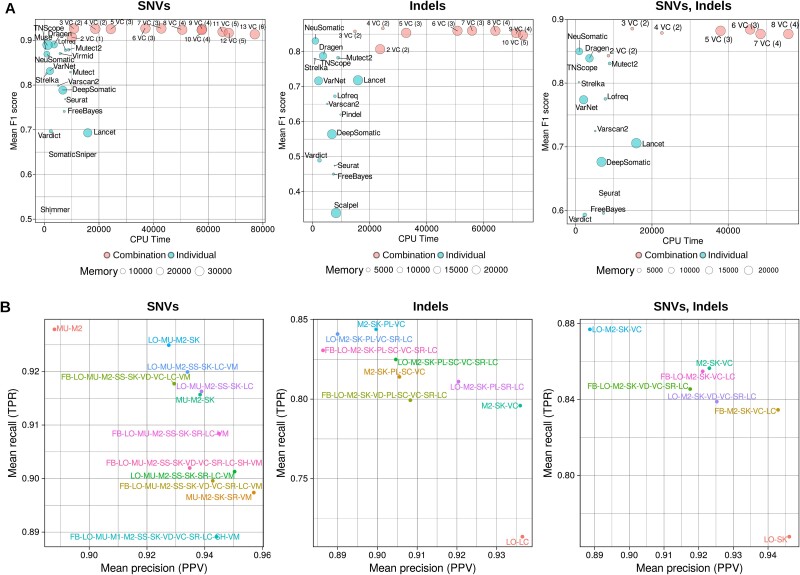
F1 scores according to computational time in the individual somatic variant callers and best ensembles. (A) Scatterplots comparing F1 score (*y*-axis) and calculation time (*x*-axis) for various somatic variant callers were drawn for SNVs, indels, and both. The circles represent individual callers and the best ensembles combining 2 to 13 tools (the number in brackets indicates the minimum voting threshold). The size of the circles corresponds to the amount of memory used by each tool/ensemble. (B) Mean recall (TPR) and precision (PPV) were calculated for the top-performing ensemble of each size across the datasets (FreeBayes: FB; Lancet: LC; LoFreq: LO; Muse: MU; Mutect: M1; Mutect2: M2; Pindel: PL; Scalpel: SC; Seurat: SR; Shimmer: SH; SomaticSniper: SS; Strelka: SK; Vardict: VD; Varscan2: VC; Virmid: VM).

The results showed significant differences between the individual somatic variant callers (Fig. 4, Table S6). For example, TNScope was 36 times faster than Lancet (7min38s versus 4h24min36s) even though Lancet was launched with more threads (8 versus 24). The average CPU time of the 20 somatic variant callers was 1h24min (min = 7min38s, max = 4h24min36s). Muse was one of the fastest tools used, but required 121 times more memory than SomaticSniper (33 395 Mb versus 274 Mb), for example.

Because the ensemble approach used several tools to call the somatic variants, the computational cost increased with the number of tools. However, after three variant callers, the gain in performance did not justify the additional computational cost. For example, for the detection of somatic SNVs, the best combination was obtained with 6 tools (F1 score = 0.927) for a total CPU time of 10h16min52s. A three-tool combination composed of Muse, Mutect2, and Strelka, requiring two agreeing votes, achieved similar results with a F1 score of 0.926 for a total CPU time of 3h1min26s. The same pattern emerged for the detection of somatic indels. A three-somatic variant callers solution based on Mutect2, Strelka, and Varscan2 with two agreeing votes achieved similar results (F1 score = 0.858) as the best results obtained by Mutect2, Strelka, Pindel, and Varscan2 (F1 score = 0.867) but in half of time (4h7min versus 6h50min). These two combinations represent the best tradeoff between performance and computational cost with a gain of 3% compared to the best individual somatic variant caller.

### Validation

To further validate our findings, we tested our proposed solutions on an independent dataset.

For the SNVs, our retained ensemble solution with Lofreq, Muse, Mutect2, SomaticSniper Strelka, and Lancet achieved an F1 score of 0.880 surpassing the best individual tool by 2.7%. The cost-effective solution using Muse, Mutect2, and Strelka obtained an F1 score of 0.875. For indels, the ensemble of Mutect2, Strelka, Pindel, and Varscan2 achieved an F1 score of 0.752 surpassing the best individual tool by 10.2%. The cost-effective solution using Mutect2, Strelka, and Varscan2 achieved an F1 score of 0.745 (Table S7).

## Discussion

In this benchmarking study, 20 individual somatic variants callers were evaluated for their performances across four reference datasets. We compared their performance against a voting-based ensemble approach that tested all tool combinations and voting thresholds. The observed variability across the datasets highlighted the known impact of several factors such as coverage and purity on variant caller performance. The synthetic NGV3 datasets and SEQC2 datasets obtained the best results, while the PERMED-01 dataset, representing the heterogeneity of real clinical cancer samples, achieved lower scores. The majority of tested tools demonstrated good performance in detecting somatic SNVs, with 11 out of 18 (61%) achieving a mean F1 score >80%. In contrast, detecting somatic indels proved more challenging, with only two tools exhibiting good results (mean F1 score > 80%) and 11 out of 15 tools attaining decent performance with a mean F1 score >60%.

However, five tools emerged as strong choices for individual calling: Dragen, Muse, Mutect2, NeuSomatic, and TNscope.

Dragen, Mutect2, and TNscope share similar characteristics. They use similar mathematical models and input parameters and can be used with a panel of normal samples (PON as described in GATK) for improved performance. In our tests, we did not use a PON with Dragen and TNscope, while Mutect2 was used with the public GATK resource bundle. In practice, users should build a PON with a sufficient number of normal samples sequenced on the same platform to increase model’s performance. However, Dragen and TNscope are commercially licensed softwares, while Mutect2 (GATK4) is fully open source. Mutect2 also had the highest CPU time consumption compared to Dragen and TNscope.

Muse represents an excellent choice for the detection of somatic SNVs, achieving performance close to Dragen and offering relatively fast analysis times (19min44s for whole-exome datasets in our benchmarks). NeuSomatic, a deep-learning algorithm, achieved excellent results, particularly in the detection of somatic indels. It outperformed the second-best caller by 2.4%.

In contrast, two other deep-learning models, DeepSomatic and VarNet, underperformed in our dataset PERMED-01. Note that pre-trained network models were used in our evaluation. These models were trained for specific settings and may not apply to all circumstances. DeepSomatic used WES from the SEQC2 consortium to build his WES pre-trained model, while VarNet used WGS data. The high contamination and error rate of the PERMED-01 dataset may have contributed to the underperformance of these models in this specific context. This situation perfectly illustrates the limitations of pre-trained deep-learning models, particularly their lack of generalizability and the difficulty in interpreting their results due to their “black box” behavior. Due to the extensive parameter space of deep-learning algorithms, training such models necessitates large amounts of data. The optimal, yet expensive, approach involves training the model on a large dataset of high-quality samples. These samples should originate from the same laboratory, and process in the same way and have their somatic variants validated with an orthogonal technology.

In contrast, ensemble approaches are easier to set up. The comprehensive exploration of all combinations and voting thresholds revealed some general trends. The performance of the ensemble approach reached a maximum between four and six somatic variant callers, after which the performance decreased. Combining a large number of different algorithms potentially adds only redundant information, providing no more information than that of a single variant caller. The same appeared with the minimum number of agreeing votes.

Our results showed that the mean F1 score reached a plateau when the number of agreeing votes approached the majority threshold. This observation suggests that the majority rule provides the best performances. Similar results were found in previous studies [19, 22]. In contrast, Trevarton and collaborators [20] suggested to use *n* − 1 callers to accept a somatic variant, where *n* is the total number of callers. We think that, with a higher minimum number of votes, the model needs more consensus between the algorithms to decide which can be problematic in the absence of clear majority. The effectiveness of ensemble approaches depends on the diversity of the combined algorithms. It is thus important to select tools based on different algorithms.

For the detection of somatic SNVs, the best performance was obtained with an ensemble combining Lofreq, Muse, Mutect2, SomaticSniper, Strelka, and Lancet with a minimum of three agreeing votes. This achieved the best average F1 score (0.927). For the detection of somatic indels, the best performance (mean F1 score = 0.867) was achieved by an ensemble combining Mutect2, Strelka, Pindel, and Varscan2 with a minimum of two agreeing votes. Compared to any individual somatic variant caller, this represents an improvement in performance of >3%. This demonstrates how an ensemble approach and a simple voting system can increase the performance of somatic variant detection. Similar findings have already been reported in previous studies [10, 20, 22].

Comparing the performance of individual variant callers and ensemble approaches across studies remains challenging. While raw data are often accessible, inconsistencies in tool parameters hinder direct result comparisons. Previous studies reported higher F1 scores for VarDict (0.794 versus 0.622), Lancet (0.941 versus 0.721), and Strelka (0.919 versus 0.839) compared to our findings [11]. Consequently, the relative ranking of callers within a comparative study holds more significance than absolute performance metrics.

With the latest Illumina platforms, such as the NovaSeq 6000, up to 500 whole exomes can be sequenced in a single run. Combining a large number of tools in the analysis pipeline increases the computational cost and extends the time it takes to deliver results to the clinic. More and more laboratories are now using cloud services to run their entire pipelines. Adding more post-alignment procedures and tools without considering CPU time and memory consumption can result in additional costs.

The industry-standard GATK Best Practices recommend two post-alignment procedures (deduplication and BQSR). While widely adopted, the impact of these steps on somatic variant callers’ performance remains unclear. Our study demonstrated that deduplication significantly enhances the performance of most somatic variant callers. PCR amplification, a common step in library preparation for WES, can introduce multiple sequencing artifacts [50]. Deduplication effectively mitigates the impact of these artifacts, improving variant calling accuracy by reducing false positives, especially in samples with high duplication rates. It is important to note that our study focused on samples with sequencing depths between 40x and 253x. For samples with very high sequencing depth (>5000x), removing duplicated reads could introduce additional biases, including inaccurate estimation of variant allele frequencies. This is particularly true for short insert size libraries where the probability of identical reads originating from different DNA fragments is non-negligible [51]. In such cases, careful consideration should be given to the potential benefits and drawbacks of deduplication. The influence of BQSR was less pronounced and varied across different somatic variant callers and datasets. While some tools benefited from BQSR, its impact was not consistently significant. For users who want to optimize processing time, BQSR might be reconsidered if specific recommendations for a particular variant caller are unavailable.

One of the principal drawbacks of the ensemble approach is the additional computational cost.

Therefore, we considered the computational cost (CPU time) of each combination to identify a solution with the best tradeoff between performances and CPU time. Our results showed that adding more than three tools did not necessarily increase the F1 score significantly (only +0.001% for SNVs and 0.01% for indels).

For somatic SNVs, the ensemble combining Muse, Mutect2, and Strelka with a minimum of two agreeing votes achieved the same performance as the previous six-tool ensemble (mean F1 score = 0.926 versus 0.927). Similarly, for somatic indels, the ensemble with Mutect2, Strelka, and Varscan2 requiring two agreeing votes achieved comparable performance (mean F1 score = 0.858 versus 867). Notably, this solution only involves four somatic variant callers altogether representing a CPU time of 4h26min24s. These represent the best tradeoff between performance and computational cost identified in our study. These findings were also validated in another dataset from the SEQC2 consortium. When summing the CPU time of individual somatic variants callers within a combination, we represented the worst-case scenario of sequential execution. However, in high-performance computing clusters (HPC), processes are often parallelized across multiple nodes. In such execution flow, the overall CPU time of a combination is typically limited by the slowest tool within the ensemble. Several factors influence the CPU time, including CPU architecture, the number of available nodes, memory size, disk type, and the number of reads.

Interestingly, the Lofreq, Mutect2, Strelka, and Vardict combination was proposed to analyze WES data [10]. In our benchmarks, this combination achieved an average F1 score of 0.897 for the somatic SNVs and 0.804 for the somatic indels. Thus, a convergence in the minimal number of tools required seems to exist between the studies, but differences remain in the choice of specific tools. The reference datasets used in each study can explain these discrepancies.

Our study displays some limitations. First, we did not explore the impact of depth, purity, and allele frequencies with the best combination of tools. Instead, we preferred to average the results across the different reference datasets to provide the most generalizable solution. Second, the reference datasets used in our study might not encompass the full spectrum of somatic variants or samples found in a laboratory setting.

Our study focused on good-quality samples. While the proposed four-tool solution demonstrated effectiveness in these conditions, its performance might be suboptimal in more complex and challenging scenarios such as those involving FFPE samples. Preliminary results using the solution based on Muse, Mutect2, Strelka, and Varscan2 on the SEQC2 FFPE reference dataset (data not shown) did not yield optimal performance, highlighting the need for dedicated benchmarks tailored to these challenging samples.

## Conclusion

This benchmarking study evaluated the performance of individual somatic variant callers and a voting-based ensemble approach for WES data. We benchmarked 20 callers across four reference datasets and compared their performance against voting-based ensemble approaches. We showed that some individual callers offer valuable solution, but ensemble approaches significantly improve the somatic variant calling. We identified an optimal combination with the best tradeoff between performance and computational cost, requiring only a combination of four callers with comparable performance to larger ensembles.

Therefore, we proposed a solution including open-source tools like Muse, Mutect2, Strelka, and VarScan2 associated with the majority rule for the most accurate and cost-effective analysis of somatic variants in WES data.

**Key Points**

- Twenty somatic variant callers were individually compared as well as 8944 different combinations of voting-based sets in four WES reference datasets. Five individual somatic variant callers, Dragen, Muse, Mutect2, NeuSomatic, and TNscope, had strong performance.
- For the detection of somatic SNVs, an ensemble combining Lofreq, Muse, Mutect2, SomaticSniper, Strelka, and Lancet with a minimum of three agreeing votes outperformed the best individual somatic variant caller by >3% (mean F1 score = 0.927).
- For the detection of somatic indels, an ensemble combining Mutect2, Strelka, Varscan2, and Pindel with a minimum of two agreeing votes outperformed the best individual somatic variant caller by >3% (mean F1 score = 0.867).
- For the best tradeoff between computational time and performances, we recommend the combination of Muse, Mutect2, and Strelka for the SNVs and Mutect2, Strelka, and Varscan2 for the indels with two agreeing votes.

## Supplementary Material

FigS1_bbae697

FigS2_bbae697

FigS3_bbae697

FigS4_bbae697

FigS5_bbae697

FigS6_bbae697

FigS7_bbae697

TableS1_bbae697

TableS2_bbae697

TableS3_bbae697

TableS4_bbae697

TableS5_bbae697

TableS6_bbae697

TableS7_bbae697

Supplementary_methods_bbae697

Supplementary_caption_bbae697

## Data Availability

All the reference datasets used in this study are publicly available. NGV3 dataset can be downloaded from https://github.com/bcbio/bcbio-nextgen. SEQC2 and HCC1143 dataset can be retrieved from SRA (https://www.ncbi.nlm.nih.gov/sra). PERMED-01 whole-exome dataset and t-NGS can be downloaded from EGA (https://ega-archive.org/studies/EGAS00001003290 and https://ega-archive.org/studies/EGAS00001004554). The pipeline to run the somatic analysis is implemented with Snakemake and is available at https://github.com/ArnaudG13/workflow-somatic, and the code to reproduce the analysis is available at https://github.com/ArnaudG13/benchmark_somatic_wes.

## Abbreviations

BQSR, base quality score recalibration; FP, false negative; FPR, false-positive rate; FN, false negative; GATK, genome analysis tool kit; HPC, high-performance computing clusters; ICGC, International Cancer Genome Consortium; INDEL, insertion/deletion; N, negative; P, positive; PPV, positive predictive value; SNV, single-nucleotide variants; SRA, Sequence Read Archive; TCGA, The Cancer Genome Atlas; TN, true negative; t-NGS, targeted next-generation sequencing; TP, true positive; TPR, true-positive rate; VAF, variant allele frequency; WES, whole-exome sequencing; WGS, whole-genome sequencing.
