# Supplementary figures and images for: A benchmarking study of individual somatic variant callers and voting-based ensembles for whole-exome sequencing
Source: Brief Bioinform. 2025 Jan 18;26(1):bbae697. doi: 10.1093/bib/bbae697 (PMC11790059; doi:10.1093/bib/bbae697)

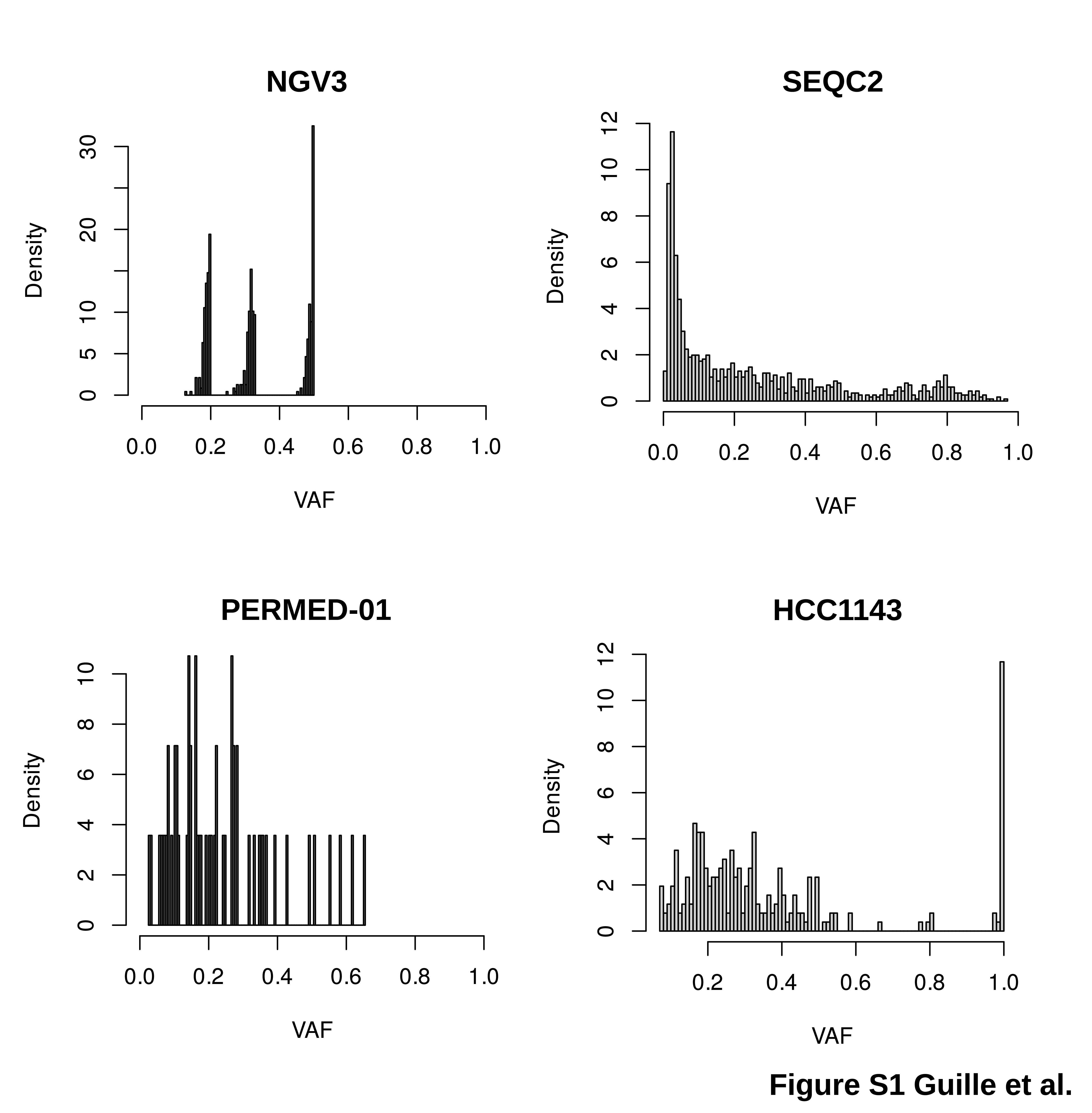

Supplement: FigS1_bbae697 [file figs1_bbae697.jpeg]

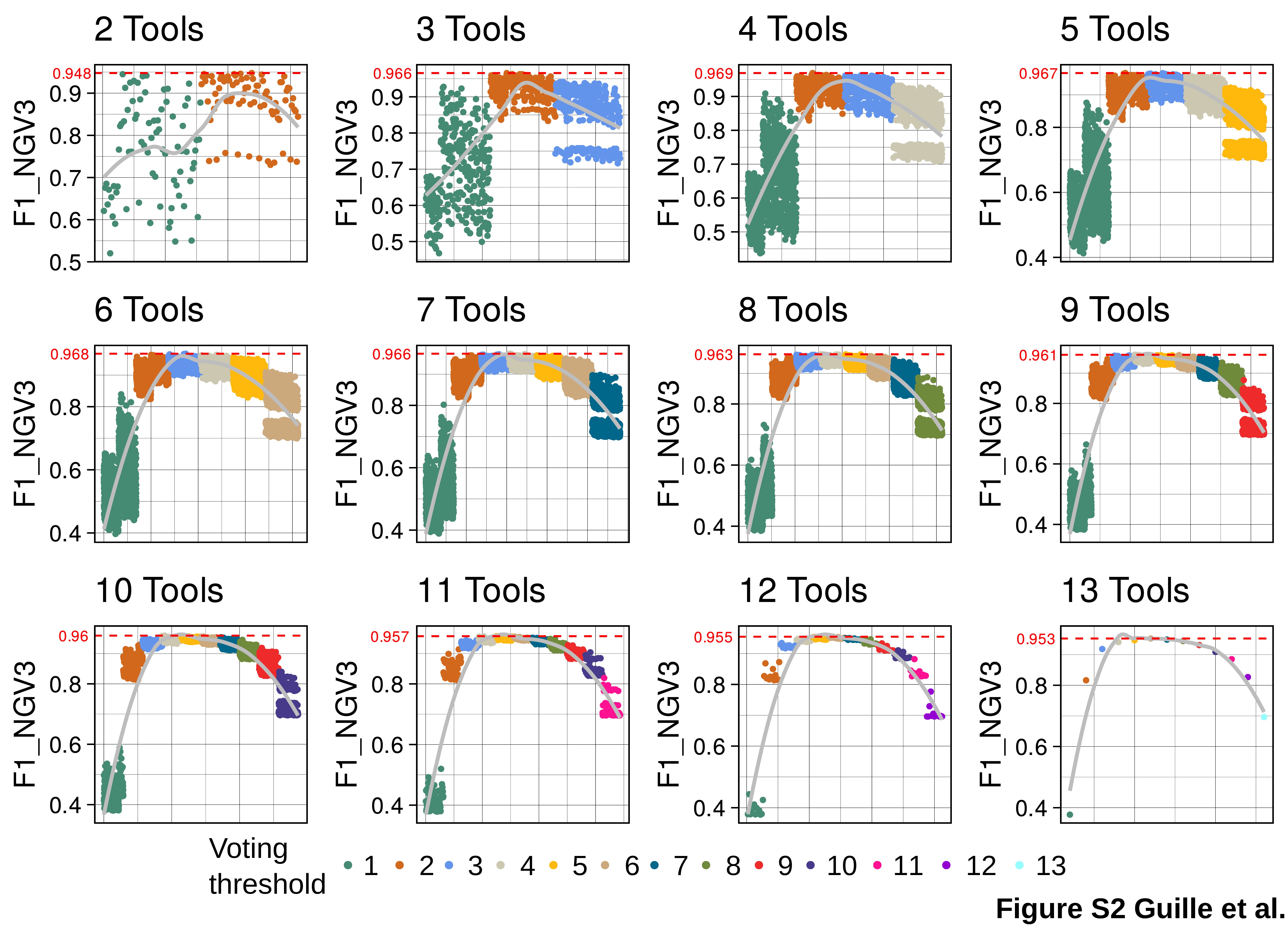

Supplement: FigS2_bbae697 [file figs2_bbae697.jpeg]

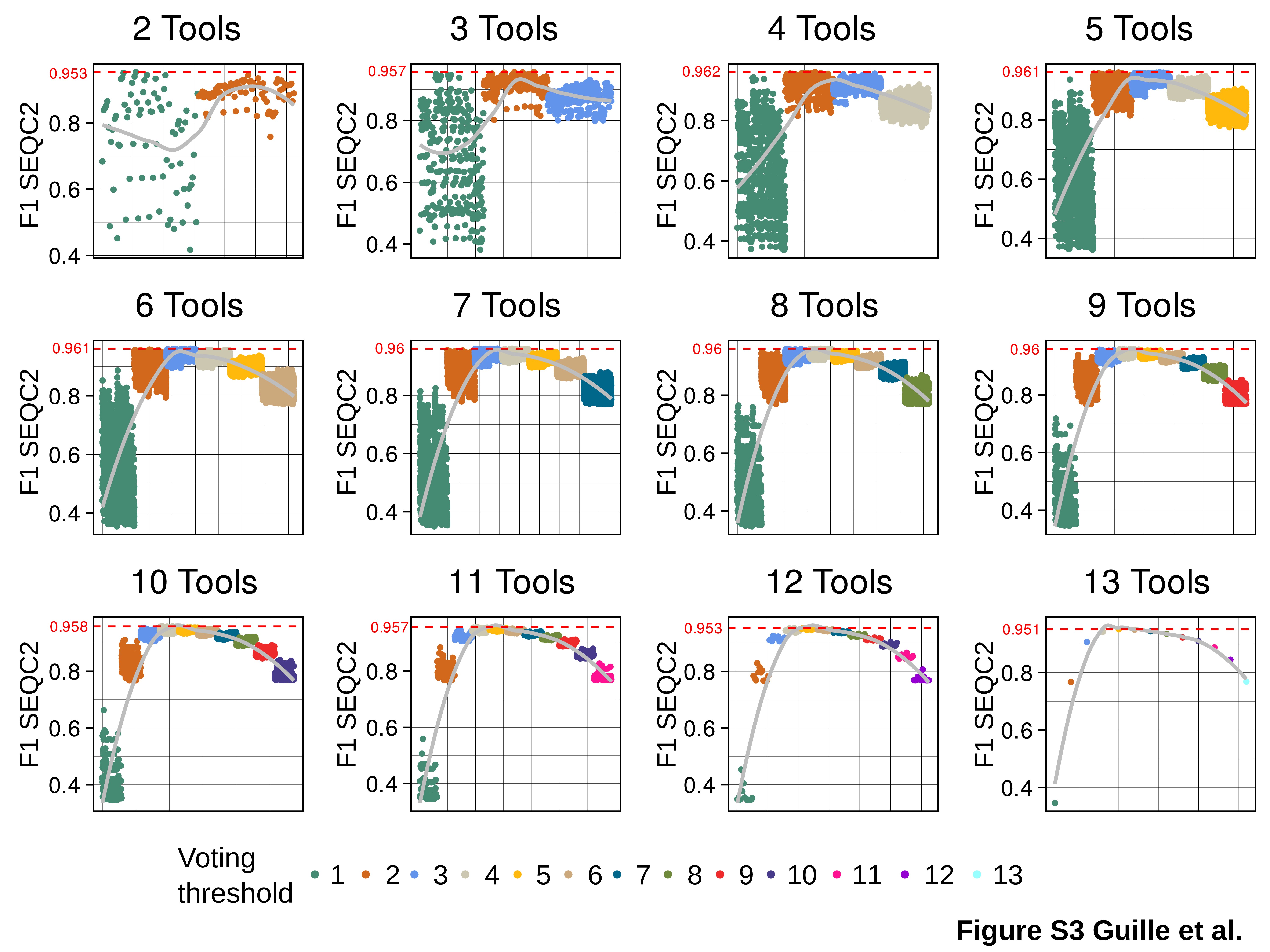

Supplement: FigS3_bbae697 [file figs3_bbae697.jpeg]

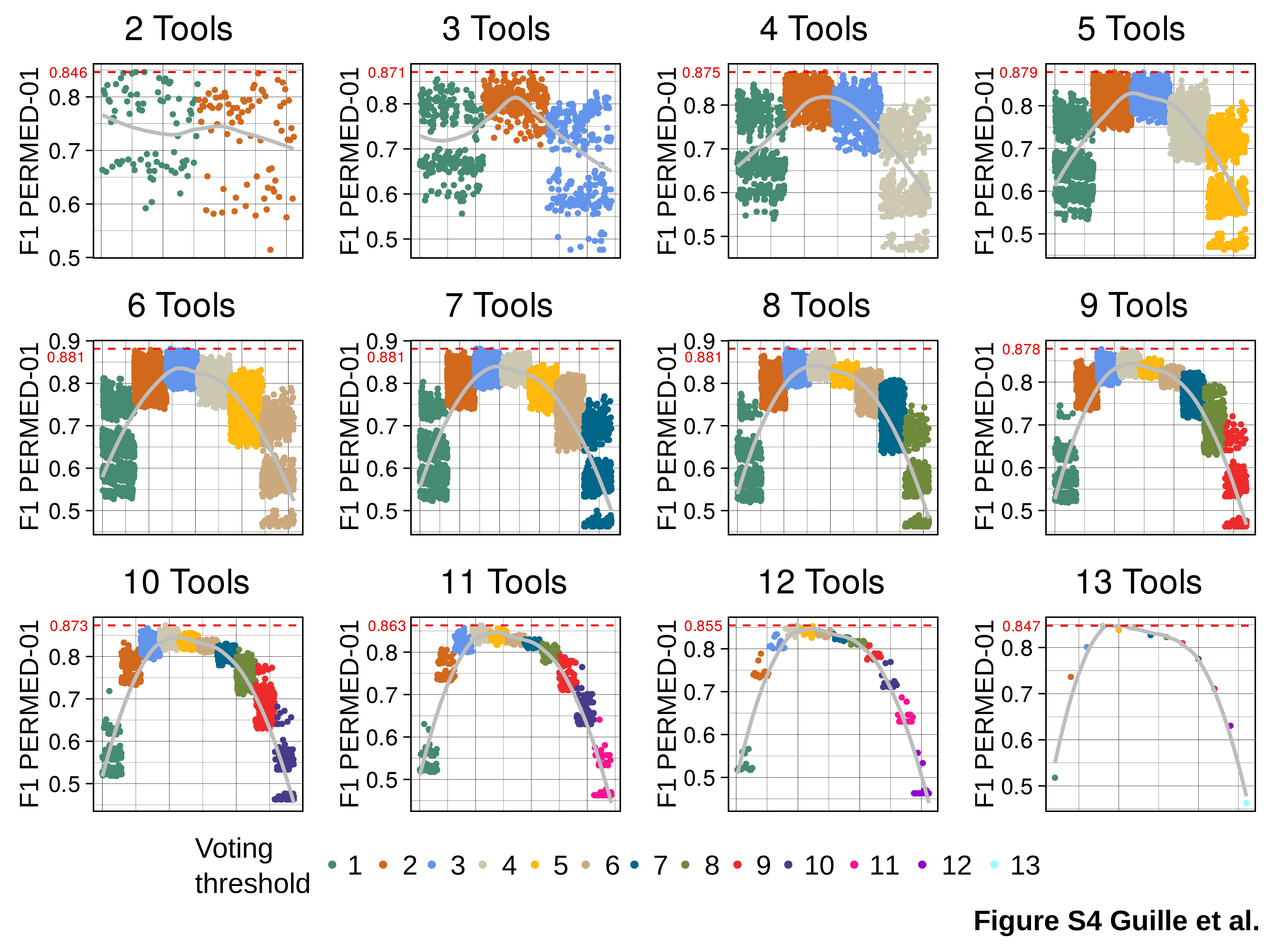

Supplement: FigS4_bbae697 [file figs4_bbae697.jpeg]

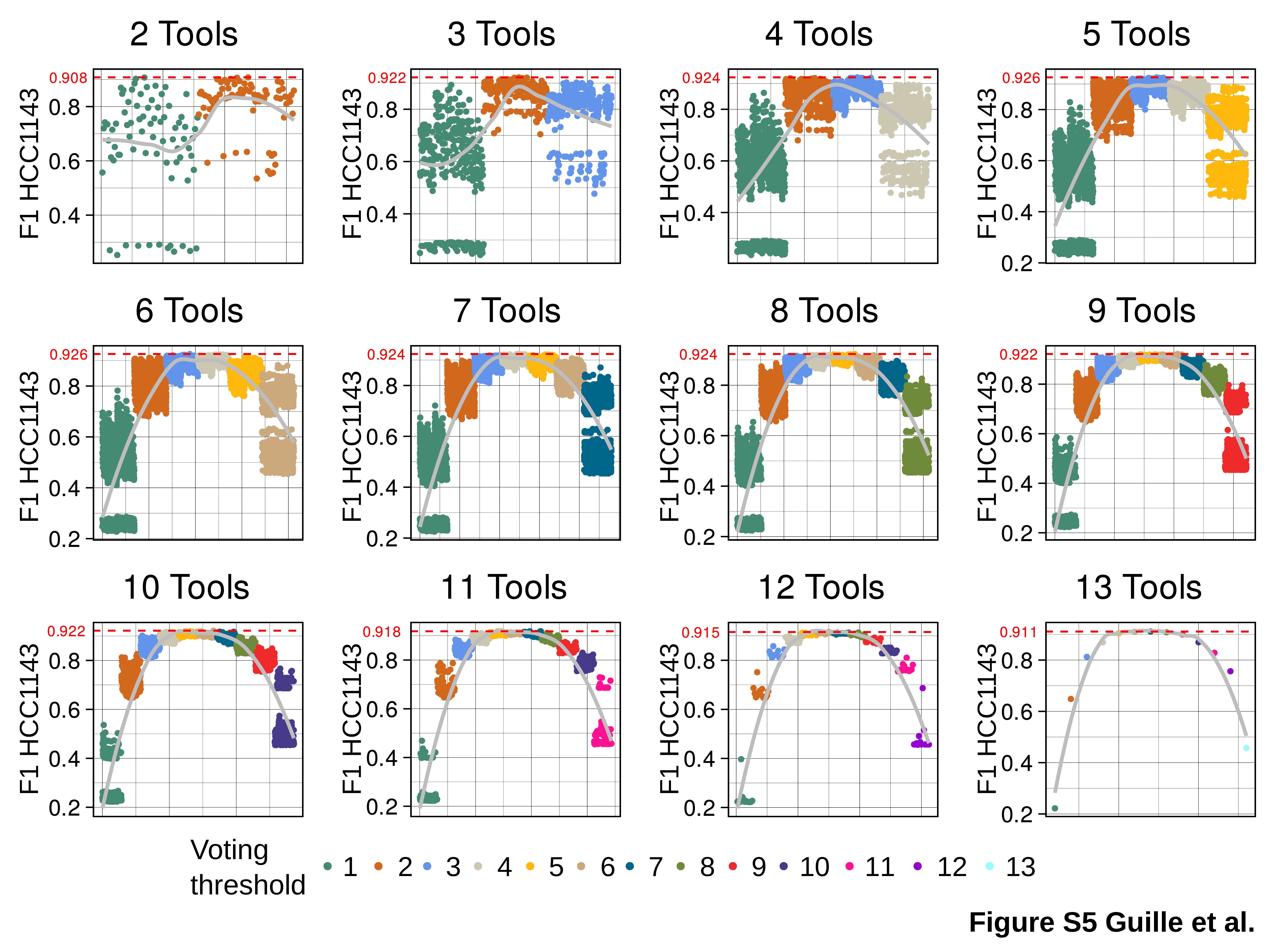

Supplement: FigS5_bbae697 [file figs5_bbae697.jpeg]

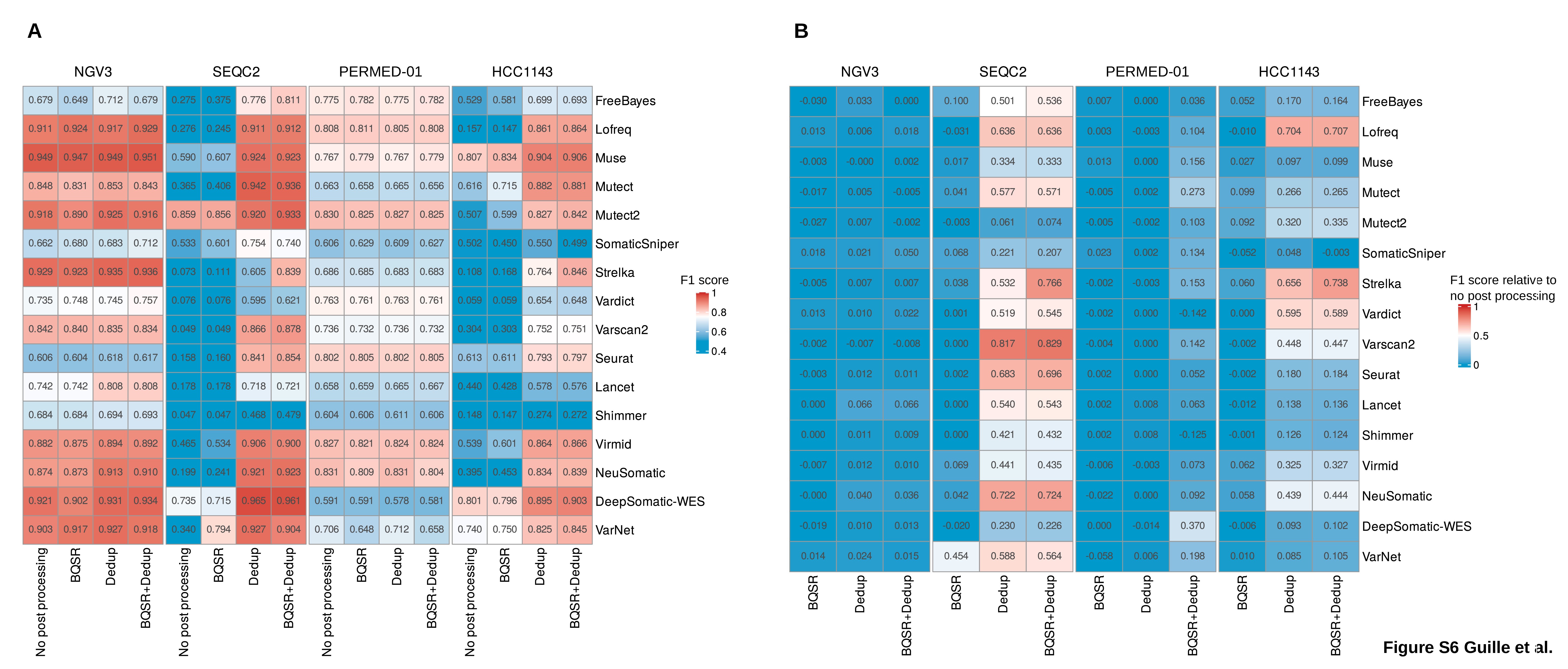

Supplement: FigS6_bbae697 [file figs6_bbae697.jpeg]

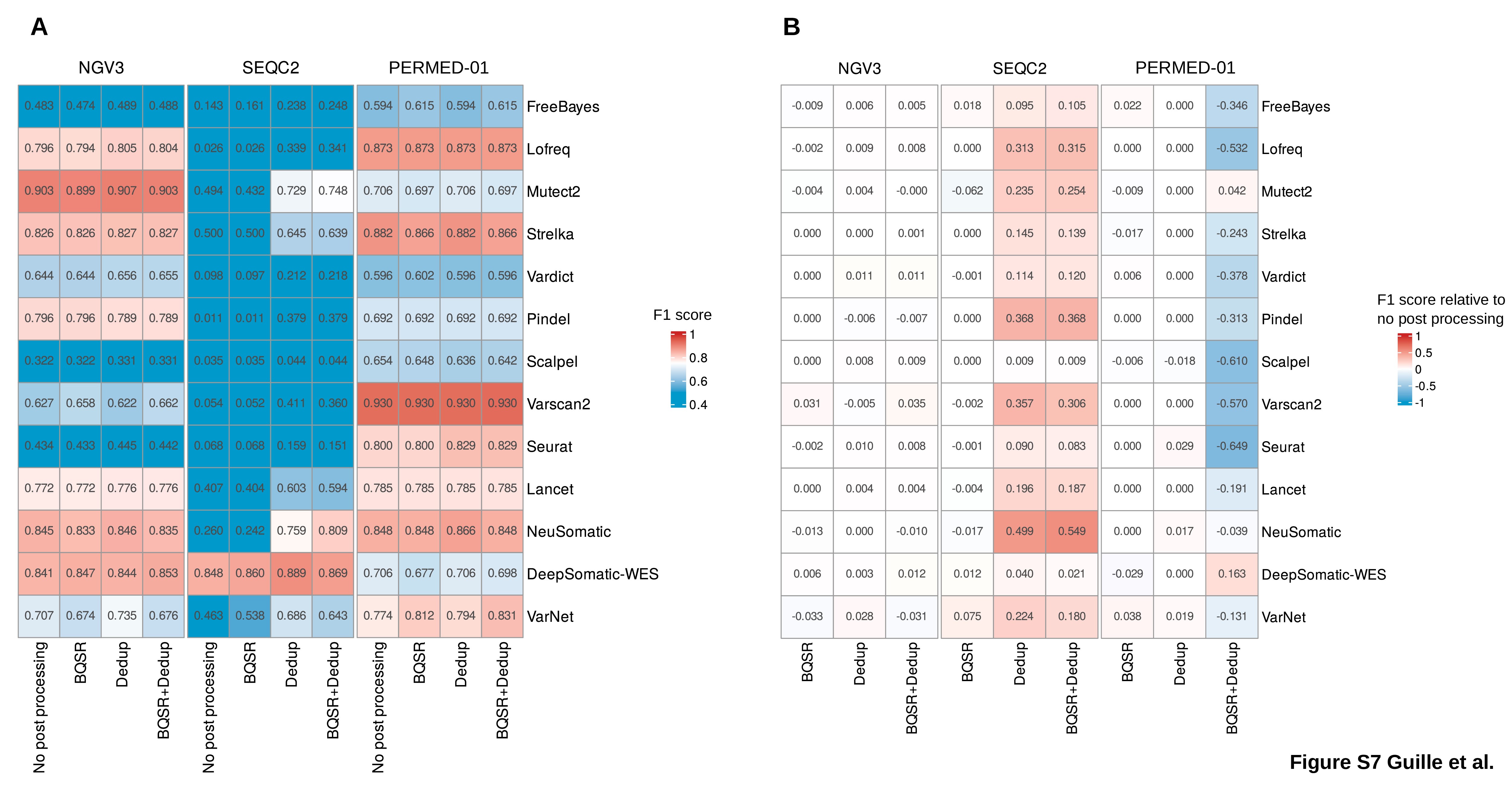

Supplement: FigS7_bbae697 [file figs7_bbae697.jpeg]
