# Supplementary material for: A benchmarking study of individual somatic variant callers and voting-based ensembles for whole-exome sequencing
Source: Brief Bioinform. 2025 Jan 18;26(1):bbae697. doi: 10.1093/bib/bbae697 (PMC11790059; doi:10.1093/bib/bbae697)
Supplement: Supplementary_methods_bbae697 [file supplementary_methods_bbae697.docx]

**Supplementary methods**

Command lines and parameters used to call the somatic variants.

#FreeBayes

freebayes -b {input.paired[0]} -b {input.paired[1]} \

-v {output} \

-t {input.target} \

-f {input.ref} \

-F 0.02 \

--min-coverage 10 \

-C 2 \

-m 30 \

-q 20 \

-R 0 \

-S 0 \

--pooled-discrete \

--pooled-continuous \

--allele-balance-priors-off

#Additional script see https://github.com/bcbio/bcbio-nextgen/blob/master/bcbio/variation/freebayes.py#L118

workflow/scripts/somatic_freebayes.py {input.vcf} $seq_name_tumor $seq_name_normal > {output}

#LoFreq

lofreq indelqual –dindel \

--ref {input.ref} \

-o normal.dindel.bam {input.paired[1]}

lofreq indelqual –dindel \

--ref {input.ref} \

-o tumor.dindel.bam {input.paired[0]}

lofreq somatic –call-indels \

-n normal.dindel.bam \

-t tumor.dindel.bam \

-f {input.ref} \

--threads 8 \

-l {input.target} \

-o {wildcards.seq_name}_vs_{wildcards.normal}.LoFreq. \

-d {input.dbsnp}

#Muse

/opt/MuSE/MuSE call \

-n 8 \

-O Muse/{wildcards.seq_name}_vs_{wildcards.normal} \

-f {input.ref} {input.paired[0]} {input.paired[1]} \

/opt/MuSE/MuSE sump -n 8 \

-I {output.txt} \

-E -O {output.vcf} \

-D {input.dbsnp}

#Mutect

java -Xmx6G -jar /usr/share/java/mutect-1.1.7.jar -T MuTect \

--reference_sequence {input.ref} \

--cosmic {input.cosmic} \

--dbsnp {input.dbsnp} \

--intervals {input.target} \

-I:tumor {input.paired[0]} \

-I:normal {input.paired[1]} \

--out {output.stats} \

--vcf {output.vcf} \

--max_alt_allele_in_normal_fraction 0.05 \

--max_alt_alleles_in_normal_count 5 \

-dfrac 1 \

--pir_median_threshold 0 \

--tumor_lod 10

#Mutect2

export OMP_NUM_THREADS={resources.cpus_per_task}

file_name=$(basename {input.paired[1]})

name=${{file_name%.sort*}}

gatk Mutect2 \

--java-options -Xmx8G \

--native-pair-hmm-threads 8 \

--reference {input.ref} \

-I {input.paired[0]} \

-I {input.paired[1]} \

-normal $name \

--germline-resource {input.gnomad} \

--genotype-germline-sites true \

--genotype-pon-sites true \

--interval-padding {params.interval_padding} \

-pon {input.pon} \

-L {input.target} \

--f1r2-tar-gz {output.f1r2} \

-O {output.vcf}

#SomaticSniper

bam-somaticsniper \

-q 40 \

-Q 15 \

-G \

-L \

-F vcf \

-f {input.ref} {input.paired[0]} {input.paired[1]} {output}

#Strelka

configureStrelkaSomaticWorkflow.py –exome \

--normal={input.paired[1]} \

--tumor={input.paired[0]} \

--ref={input.ref} \

--runDir=tmp \

--callRegions {input.target}.gz

#VarDict

VarDict

–dedup \

-th 8 \

-G {input.ref} \

-f 0.02 \

-N {wildcards.seq_name} \

-r 3 \

-b "{input.paired[0]}|{input.paired[1]}" \

-c 1 -S 2 -E 3 -g 4 {input.target} | testsomatic.R | var2vcf_paired.pl -N "{wildcards.seq_name}|{wildcards.normal}" -f 0.02 > {output}

#Pindel

pindel \

-T 4 \

-f {input.ref} \

-i {input.config} \

--chromosome {wildcards.chr} \

-w 10 -M 5 -o tmp/{wildcards.chr}.INDEL

#Scalpel

/opt/scalpel-0.5.4/scalpel-discovery \

--somatic \

--normal {input.paired[1]} \

--tumor {input.paired[0]} \

--bed {input.target} \

--ref {input.ref} \

--dir tmp \

--numprocs 8 \

--intarget

/opt/scalpel-0.5.4/scalpel-export \

--somatic \

--db tmp/main/somatic.db.dir \

--bed {input.target} \

--ref {input.ref} \

--min-alt-count-tumor 2 \

--min-vaf-tumor 0.02 \

--min-coverage-tumor 10 \

--intarget > Scalpel.somatic.indel.vcf

#Varscan2

samtools mpileup -ABQ0 \

-q 15 \

-d 100000 \

-f {input.ref} \

-l {input.target} \

{input.paired[1]} {input.paired[0]} | awk '{{if($4 != 0) print $0}}' | awk '{{if($7 != 0) print $0}}' | java -Xmx2G -jar /usr/share/java/VarScan.v2.3.9.jar somatic -mpileup VarScan2.somatic \

--output-vcf 1 \

--min-coverage 10 \

--min-reads2 2 \

--min-avg-qual 20 \

--min-var-freq 0.02 \

--strand-filter 0

#Seurat

java -Xmx2G -jar /usr/share/java/Seurat-2.5.jar -T Seurat \

-R {input.ref} \

-I:dna_normal {input.paired[1]} \

-I:dna_tumor {input.paired[0]} \

-L {input.target} \

--indels \

-Q 15 \

-insert_size 200 \

-mmq 15 \

-mbq 20 \

-mcv 6 \

-mm 3 \

-o {output} \

-go tmp.txt

#Lancet

lancet \

--tumor {input.paired[0]} \

--normal {input.paired[1]} \

--ref {input.ref} \

--min-alt-count-tumor 3 \

--max-alt-count-normal 1 \

--min-vaf-tumor 0.02 \

--max-vaf-normal 0.01 \

--min-coverage-tumor 4 \

--min-coverage-normal 10 \

--bed {input.target} \

--num-threads 8 > {output}

#Shimmer

shimmer.pl \

--bedfile {input.target} \

--ref {input.ref} \

--minqual 15 \

--mapqual 10 \

--max_q 0.25 \

--outdir {wildcards.seq_name} \

{input.paired[1]} \

{input.paired[0]}

#Virmid

java -Xmx16G -jar /opt/virmid-1.1.0/Virmid.jar \

-R {input.ref} \

-D {input.paired[0]} \

-N {input.paired[1]} \

-w Virmid \

-r 150 \

-t 8 \

-c1 10 \

-C1 1000 \

-c2 10 \

-C2 1000 \

-o {wildcards.seq_name}

#NeuSomatic

python /opt/neusomatic/neusomatic/python/preprocess.py --mode call \

--reference {input.ref} \

--region_bed {input.target} \

--tumor_bam {input.paired[0]} \

--normal_bam {input.paired[1]} \

--work work_call \

--dbsnp_to_filter {input.dbsnp} \

--scan_maf 0.01 \

--snp_min_af 0.03 \

--snp_min_bq 15 \

--snp_min_ao 3 \

--ins_min_af 0.02 \

--del_min_af 0.02 \

--min_mapq 10 \

--num_threads 20 \

--scan_window_size 500 \

--max_dp 100000 \

--scan_alignments_binary /opt/neusomatic/neusomatic/bin/scan_alignments

python /opt/neusomatic/neusomatic/python/call.py \

--candidates_tsv {params.base_dir}/{wildcards.sample}/SomaticAnalysis/NeuSomatic/work_call/dataset/*/candidates*.tsv \

--reference {input.ref} \

--out {params.base_dir}/{wildcards.sample}/SomaticAnalysis/NeuSomatic/work_call \

--checkpoint /opt/neusomatic/neusomatic/models/NeuSomatic_v0.1.4_standalone_SEQC-WGS-GT50-SpikeWGS10.pth \

--num_threads 20 \

--batch_size 100

python /opt/neusomatic/neusomatic/python/postprocess.py \

--reference {input.ref} \

--tumor_bam {input.paired[0]} \

--pred_vcf work_call/pred.vcf \

--candidates_vcf work_call/work_tumor/filtered_candidates.vcf \

--output_vcf NeuSomatic.somatic.vcf \

--work work_call

#DeepSomatic

run_deepsomatic \

--model_type=WGS \

--ref={input.ref} \

--reads_normal={input.paired[1]} \

--reads_tumor={input.paired[0]} \

--output_vcf=DeepSomatic.somatic.vcf.gz \

--sample_name_tumor="{wildcards.seq_name}" \

--sample_name_normal="{wildcards.normal}" \

--num_shards=20 \

--logging_dir=DeepSomatic \

--intermediate_results_dir=DeepSomatic/tmp \

--regions={input.target}

#VarNet

python /VarNet/filter.py \

--sample_name {wildcards.seq_name} \

--normal_bam {input.paired[1]} \

--tumor_bam {input.paired[0]} \

--processes 8 \

--output_dir VarNet \

--reference {input.ref} \

--region_bed {input.target}

python /VarNet/predict.py \

--sample_name {wildcards.seq_name} \

--normal_bam {input.paired[1]} \

--tumor_bam {input.paired[0]} \

--processes 8 \

--output_dir VarNet \

--reference {input.ref}

#TNScope

sentieon-genomics-202308.02/bin/sentieon driver \

-t 8 \

-r {input.ref} \

-i {input.paired[0]} \

-i {input.paired[1]} \

--algo TNscope \

--tumor_sample {wildcards.seq_name} \

--normal_sample {wildcards.normal} \

--dbsnp {input.dbsnp} \

--pon {input.pon} \

--min_tumor_lod 10 \

{output}
