# Supplementary material for: A benchmarking study of individual somatic variant callers and voting-based ensembles for whole-exome sequencing
Source: Brief Bioinform. 2025 Jan 18;26(1):bbae697. doi: 10.1093/bib/bbae697 (PMC11790059; doi:10.1093/bib/bbae697)
Supplement: Supplementary_caption_bbae697 [file supplementary_caption_bbae697.docx]

**Figure S1.**

Distribution of the somatic variant allele frequency (VAF) in the four datasets.

Alt text: Graphs depicting the distribution of the somatic variant allele frequency in the four datasets.

**Figure S2.**

Performance of all somatic variant callers combinations in NGV3 dataset. This analysis evaluated performance with all possible combinations of somatic variant callers, ranging from 2 to 13 tools, for SNV detection. Results are visualized in a scatter plot, where each point represents a unique ensemble. Colors represent the voting threshold used within the ensemble. The y-axis reflects the performance (F1 score) of each ensemble. The red dashed line exhibits the maximum F1 score.

Alt text: Graphs depicting the performance of all somatic variant callers combinations by voting threshold and number of tools, in NGV3 dataset.

**Figure S3.**

Performance of all somatic variant callers combinations in SEQC2 dataset. This analysis evaluated performance with all possible combinations of somatic variant callers, ranging from 2 to 13 tools, for SNV detection in the SEQC2 dataset. The results are visualized in a scatter plot, where each point represents a unique ensemble. Colors represent the voting threshold used within the ensemble. The y-axis reflects the performance (F1 score) of each ensemble. The red dashed line exhibits the maximum F1 score.

Alt text: Graphs depicting the performance of all somatic variant callers combinations by voting threshold and number of tools, in SEQC2 dataset.

**Figure S4.**

Performance of all somatic variant callers combinations in PERMED-01 dataset. This analysis evaluated performance with all possible combinations of somatic variant callers, ranging from 2 to 13 tools, for SNV detection in the PERMED-01 dataset. The results are visualized in a scatter plot, where each point represents a unique ensemble. Colors represent the voting threshold used within the ensemble. The y-axis reflects the performance (F1 score) of each ensemble. The red dashed line exhibits the maximum F1 score.

Alt text: Graphs depicting the performance of all somatic variant callers combinations by voting threshold and number of tools, in PERMED-01 dataset.

**Figure S5.**

Performance of all somatic variant callers combinations in HCC1143 dataset. This analysis evaluated performance with all possible combinations of somatic variant callers, ranging from 2 to 13 tools, for SNV detection in the HCC1143 dataset. The results are visualized in a scatter plot, where each point represents a unique ensemble. Colors represent the voting threshold used within the ensemble. The y-axis reflects the performance (F1 score) of each ensemble. The red dashed line exhibits the maximum F1 score.

Alt text: Graphs depicting the performance of all somatic variant callers combinations by voting threshold and number of tools, in HCC1143 dataset.

**Figure S6.**

Influence of the post-alignments procedure on the performances of the somatic variant calling for SNVs.

(A) Heatmap of F1 score for the SNVs across datasets and post-alignment procedures. (B) Heatmap of the relative F1 score across datasets for each post-alignment procedures. The reference for the comparisons was no post-alignment.

Alt text: Heatmap depicting the performance of all the somatic variant callers for the SNVs across four datasets and according to the post-alignment procedures.

**Figure S7.**

Influence of the post-alignments procedure on the performances of the somatic variant calling for indels.

(A) Heatmap of F1 score for the SNVs across datasets and post-alignment procedures. (B) Heatmap of the relative F1 score across datasets for each post-alignment procedures. The reference for the comparisons was no post-alignment.

Alt text: Heatmap depicting the performance of all the somatic variant callers for the indels across three datasets and according to the post-alignment procedures.

**Supplementary Tables**

Table S1. Performance of individual somatic variant callers for SNVs detection across datasets and post-alignment procedures

Table S2. Performance of individual somatic variant callers for indels detection across datasets and post-alignment procedures.

Table S3. Performance of all combinations of somatic variant callers for SNVs detection.

Table S4. Performance of all combinations of somatic variant callers for indels detection.

Table S5. Performance of all combinations of somatic variant callers for both SNVs and indels detection.

Table S6. Performance and computational time of individual somatic variant callers and best ensembles.

Table S7. Performance of individual somatic variant callers and retained best combinations in the validation dataset.
